# Immune response, antibody persistence, and safety of a single dose of the quadrivalent meningococcal serogroups A, C, W-135, and Y tetanus toxoid conjugate vaccine in adolescents and adults: results of an open, randomised, controlled study

**DOI:** 10.1186/1471-2334-13-116

**Published:** 2013-03-05

**Authors:** Charissa Borja-Tabora, Cecilia Montalban, Ziad A Memish, Marie Van der Wielen, Veronique Bianco, Dominique Boutriau, Jacqueline Miller

**Affiliations:** 1 Research Institute for Tropical Medicine, Muntinlupa City, Philippines; 2 Philippine General Hospital Manila, Manila, Philippines; 3 College of Medicine, Alfaisal University, PO Box 54146, Riyadh, 11514, Saudi Arabia; 4 GlaxoSmithKline Vaccines, Wavre, Belgium; 5 GlaxoSmithKline Vaccines, King of Prussia, PA, USA

**Keywords:** Quadrivalent meningococcal vaccine, Conjugate vaccine, Bactericidal activity, Persistence, Safety, The Philippines, Saudi Arabia

## Abstract

**Background:**

The best strategy to protect individuals against meningococcal disease is to immunize against multiple serogroups. Immunogenicity, antibody persistence, and safety of the EU-licensed meningococcal ACWY-tetanus toxoid conjugate vaccine (MenACWY-TT) were evaluated in healthy participants aged 11–55 years from the Philippines and Saudi Arabia.

**Methods:**

In this phase IIb, open, controlled study, 500 participants were randomised (3:1) to receive one dose of MenACWY-TT or a licensed meningococcal polysaccharide vaccine (Men-PS). Functional antibody responses against meningococcal serogroups A, C, W-135, and Y were assessed by a serum bactericidal antibody assay using rabbit complement (rSBA) at Month 0, Month 1, Year 1, Year 2, and Year 3. Vaccine response was defined as an rSBA titre ≥32 at Month 1 in participants who were seronegative (rSBA titre <8) pre-vaccination and as at least a four-fold increase in titre in participants who were seropositive pre-vaccination. Solicited symptoms were recorded up to Day 4, safety outcomes up to Month 6, and serious adverse events related to vaccination up to Year 3.

**Results:**

Pre-specified criteria for non-inferiority of MenACWY-TT versus Men-PS were met in terms of rSBA vaccine response and incidence of grade 3 general symptoms. At Month 1, 82.7%–96.3% of MenACWY-TT and 69.7%–91.7% in Men-PS recipients had a vaccine response for each serogroup. At Year 3, ≥99.1% and ≥92.9% of MenACWY-TT recipients retained rSBA titres ≥8 and ≥128, respectively, as compared to ≥86.7% and ≥80.0% in the Men-PS group. Both vaccines had a clinically acceptable safety profile, although injection site redness and swelling were more frequent in MenACWY-TT recipients.

**Conclusions:**

These results suggest that MenACWY-TT could protect adolescents and adults against meningococcal disease up to three years post-vaccination.

**Trial registration:**

This study is registered at http://www.clinicaltrials.gov/NCT00356369.

## Background

*Neisseria meningitidis* is a major cause of endemic and epidemic invasive bacterial disease worldwide and is associated with high morbidity [1-3]. Most invasive meningococcal disease is caused by six of the 12 known serogroups, namely serogroups A, B, C, W-135, Y, and more recently X [2,3]. The epidemiology of meningococcal disease varies geographically and over time, and the increasing trend of international travel and migration facilitates the rapid intercontinental spread of meningococcal isolates [4-7]. Moreover, new strains may also appear in a region by bacterial capsular switching [3,8].

In Asia and the Middle East, serogroups A, C, and W-135 are the most common serogroups [3,5,9]. In the Philippines, an outbreak caused by serogroup A occurred in 2004–2005 [3,10]. A previous outbreak had been reported in 1989, but data on serogroup distribution from this earlier outbreak are not available [11]. In Saudi Arabia, *N*. *meningitidis* has been responsible for various outbreaks during the annual Hajj pilgrimage [12,13]. An outbreak caused by serogroup A occurred in 1987 and an outbreak caused by serogroup W-135 was also reported in 2000–2001 [12-15]. Pilgrims to the Hajj are at increased risk for infection because of overcrowding, shared living facilities, and close contact with people from various parts of the world, which are all known risk factors for meningococcal disease [13].

Immunisation with vaccines against multiple serogroups is likely to be the best strategy to protect individuals against meningococcal diseases. In both the Philippines and Saudi Arabia, vaccination is recommended for individuals who are at increased risk for meningococcal infection. In particular, vaccination is used to protect Hajj pilgrims against invasive meningococcal disease in Saudi Arabia [5,12]. In response to the 1987 outbreak, vaccination with a bivalent polysaccharide vaccine against serogroups A and C was recommended for Hajj pilgrims, and the vaccination policy was updated following the outbreak in 2000–2001 to include serogroup W-135 [7]. Presently, Saudi Arabia requires proof of vaccination with a quadrivalent meningococcal serogroup A, C, W-135, and Y vaccine to issue Hajj pilgrimage visa for all arrivals. Moreover, administration of chemoprophylaxis at the port of entry to all arrivals from the countries of the African meningitis belt is required in order to lower the carriage rate among Hajj pilgrims [15,16].

Plain meningococcal polysaccharide vaccines against serogroups A, C, W-135, and Y are available for use in adults and children over two years of age. However, these vaccines are poorly immunogenic for serogroups C, W-135 and Y, do not elicit long-term protection in younger children, do not induce immune memory, do not reduce mucosal carriage (except for serogroup A), and do not confer herd protection [17-20]. Moreover, polysaccharide vaccines may induce hyporesponsiveness to subsequent vaccination, in particular for serogroup C [17]. To overcome these limitations, capsular polysaccharides can be coupled to carrier proteins as demonstrated by monovalent meningococcal serogroup C conjugate vaccines [21-24]. In 2010, the Ministry of Health in Saudi Arabia decided to replace the meningococcal polysaccharide vaccines by meningococcal conjugate vaccines for Saudi Arabian pilgrims, residents of Medina and Mecca Regions, and healthcare workers [12,13]. The introduction of conjugate vaccines, which have an impact on carriage and transmission of the bacteria, could reduce the spread of isolates from asymptomatic pilgrims to their household contacts upon returning from the pilgrimage [6,14].

Presently, two quadrivalent meningococcal conjugate vaccines against serogroups A, C, W-135, and Y are licensed in various countries [25-27]. Additionally, a new quadrivalent meningococcal serogroups A, C, W-135, and Y tetanus-toxoid (TT) conjugate vaccine (*Nimenrix*™ [GlaxoSmithKline Vaccines]; MenACWY-TT) has been recently approved by the European Medicines Agency for individuals aged 12 months and above. Previous studies have shown that MenACWY-TT is immunogenic and well-tolerated in toddlers, young children, adolescents, and young adults [28-34]. In the present study, we compared the immunogenicity and safety of MenACWY-TT and a licensed quadrivalent meningococcal serogroups A, C, W-135, and Y plain polysaccharide vaccine in participants 11–55 years of age in the Philippines and Saudi Arabia. The study was designed to evaluate the persistence of the immune response up to five years after vaccination; results up to the third year were available at the time of the preparation of this manuscript.

## Methods

### Study design

This was a phase IIb, open, randomised, controlled study conducted in two centres in the Philippines (Research Institute for Tropical Medicine and Phillippine General Hospital) and one centre in Saudi Arabia (King Abdulaziz Medical City in Riyadh). The study consisted of two phases: the vaccination phase (including the extended safety follow-up until Month 6) and the long-term persistence phase (up to five years after vaccination). At the time of the preparation of this manuscript, results up to the third year post-vaccination were available (covering the period December 2006 to August 2010).

Participants were randomised (3:1) into two parallel groups to receive either one dose of the MenACWY-TT vaccine (ACWY-TT group) or one dose of the MenACWY polysaccharide vaccine (Men-PS group) at Month 0. The randomisation list was generated at GlaxoSmithKline (Rixensart, Belgium) using a standard Statistical Analysis System (SAS®) program to number the vaccines. Treatment allocation at the investigator sites was performed using a central internet randomisation system. The randomisation algorithm used a minimisation procedure (block size of four) accounting for study centre and age of the subject (11–17 years and 18–55 years of age) in order to ensure a balanced distribution of the population in each group (2/3 of subjects were enrolled in the 11–17 year age stratum, and 1/3 of subjects were enrolled in the 18–55 year age stratum). The vaccines were administered in an open manner due to the differing appearance and route of administration of the study vaccines.

The study was conducted in accordance with the Good Clinical Practice Guidelines and the Declaration of Helsinki. The protocol and associated documents were reviewed and approved by local ethics committees (the Institutional Review Board from the National Guard Health Affair, Riyadh, Saudi Arabia and from the Research Institute for Tropical Medicine, Muntinlupa City, the University of the Philippines Manila Research Ethics Board (UPMREB), Manila, the Philippines). Written informed consent was obtained before enrolment from the participants aged at least 16 years in Saudi Arabia and 18 years in the Philippines. Written informed consent was obtained from the parents/guardians of younger participants, and a written informed assent was signed by these participants. In addition, consent was obtained if participants in the Philippines reached 18 years of age during the study. This study is registered at http://www.clinicaltrials.gov NCT00356369. A summary of the protocol is available at http://www.gsk-clinicalstudyregister.com (GSK study ID 107386).

### Study objectives

The primary objectives of this study were to demonstrate non-inferiority of the MenACWY-TT vaccine over the MenACWY polysaccharide vaccine for the entire population in terms of vaccine response (measured by a serum bactericidal antibody assay using baby rabbit serum as exogenous complement source [rSBA]) and incidence of any grade 3 general symptom reported within four days after vaccination.

The secondary objectives of the study were to compare the immunogenicity and the persistence of the immune response of the MenACWY-TT vaccine with that of the MenACWY polysaccharide vaccine and to evaluate the reactogenicity and the safety of both vaccines.

### Study participants

Study participants were healthy adolescents and adults from the Philippines and Saudi Arabia aged 11 to 55 years at the time of vaccination, who had previously completed routine childhood vaccinations to the best of their, or their parents’/guardians’, knowledge.

Participants were excluded if they were immunosuppressed from any cause or were previously vaccinated with a meningococcal conjugate vaccine at any time or with a meningococcal polysaccharide vaccine against serogroup A, C, W-135, or Y within five years prior to the study. Participants with a history of meningococcal disease due to serogroup A, C, W-135, or Y; Guillain-Barré syndrome; any neurological disorders or seizures; allergic disease or reactions likely to be exacerbated by any component of the vaccine; or chronic alcohol consumption or drug abuse were also excluded. Moreover, participants were ineligible if they had used an investigational product within 30 days prior to the study, had received immunoglobulins or blood products in the preceding three months, or had a major congenital defect, serious illness, or acute disease at the time of enrolment. Females of childbearing potential were required to practice adequate contraception for 30 days prior to vaccination, have a negative pregnancy test at the time of vaccination, and continue contraceptive precautions for two months after vaccination.

### Vaccines

One dose of the MenACWY-TT candidate vaccine (*Nimenrix*™, GlaxoSmithKline, Rixensart, Belgium) contained 5 μg of each of the meningococcal serogroups A, C, W-135, and Y polysaccharides individually conjugated to TT. A single MenACWY-TT vaccine dose was administered intramuscularly in the non-dominant deltoid of the participants in the ACWY-TT group. One dose of the MenACWY polysaccharide vaccine (*Mencevax*™ ACWY, GlaxoSmithKline, Rixensart, Belgium) contained 50 μg of each of the meningococcal serogroups A, C, W-135, and Y polysaccharides. A single MenACWY polysaccharide vaccine dose was administered subcutaneously in the non-dominant upper arm of the participants in the Men-PS group.

### Immunogenicity assessment

Blood samples were collected from all participants at Month 0, Month 1, Year 1, Year 2, and Year 3. Functional antibody responses against meningococcal serogroups A, C, W-135, and Y were assessed by rSBA [35]. The target strains used in the rSBA assays were L10 for serogroup A, C11 for serogroup C, MP01240070 for serogroup W-135, and S-1975 for serogroup Y. rSBA titres ≥8 for serogroup C are considered to predict protection [36], and this threshold was extended to the other serogroups as well [37]. rSBA titres ≥128, a more conservative correlate of protection, were also evaluated [38]. Vaccine response was defined as an rSBA titre ≥32 at Month 1 in participants who were seronegative (rSBA titre <8) at pre-vaccination and as at least a four-fold increase in titre from Month 0 to Month 1 in participants who were seropositive at pre-vaccination.

In addition, antibody concentrations against TT were determined by enzyme-linked immunosorbent assay (ELISA) with an assay cut-off of 0.1 IU/mL [39]. All assays were performed at GlaxoSmithKline’s central laboratory (Rixensart, Belgium), and laboratory personnel were blinded to the treatment group of the subject.

### Safety and reactogenicity assessment

Local (pain, redness, and swelling) and general (fatigue, fever, gastrointestinal symptoms, and headache) solicited symptoms were recorded during four days and unsolicited adverse events (AEs) during one month after vaccination. The intensity of each symptom was graded on a three-level scale. Injection site redness and swelling were of grade 3 intensity if their diameter was >50 mm and fever if oral temperature was >39.5°C. All other symptoms of grade 3 intensity were defined as symptoms preventing normal activity.

New onset of chronic illnesses (NOCIs) and serious adverse events (SAEs) were recorded for up to six months post-vaccination. SAEs were defined as any medical event resulting in death, any life-threatening event, any event causing disability or requiring hospitalisation or prolongation of hospitalisation, or any congenital anomaly or birth defect in the offspring of a study participant. Moreover, SAEs related to vaccination and any events related to lack of vaccine efficacy were reported throughout the study. All solicited local reactions were considered causally related to vaccination. Causality of all other AEs was assessed by the investigators at the local study sites (independent from the Sponsor of the study).

### Statistical analyses

With a target sample size of 460 participants evaluable for immunogenicity (345 in the ACWY-TT group and 115 in the Men-PS group), the global power to meet all the primary objectives of the study was at least 76%.

The total vaccinated cohort, on which the safety analyses were performed, included all vaccinated participants. The analyses of immunogenicity were performed on the according to protocol (ATP) immunogenicity cohort and on the ATP cohort for antibody persistence Year 1, Year 2, and Year 3, which included all evaluable study participants, who were vaccinated during the vaccination phase, meeting the eligibility criteria, with no elimination criteria during the study, and for whom data concerning immunogenicity endpoints were available for at least one tested antigen at Month 1, Year 1, Year 2, or Year 3, respectively.

The percentages of participants with antibody titres or concentrations above the proposed cut-offs and with a vaccine response as well as the geometric mean antibody titres (GMTs) or geometric mean antibody concentrations (GMCs) for the tested antigens were calculated with 95% confidence intervals (CIs) in each treatment group. The GMTs/GMCs were calculated by taking the anti-log of the mean of the log_10_ titre/concentration transformations. Antibody titres/concentrations below the cut-off of the assay were given an arbitrary value of half the cut-off for the purpose of GMTs/GMCs calculation.

Non-inferiority of the rSBA vaccine response induced by the MenACWY-TT vaccine over the MenACWY polysaccharide vaccine was demonstrated if the lower limit (LL) of the asymptotic standardised 95% CI for the difference between the ACWY-TT and the Men-PS groups in the percentage of participants with rSBA vaccine response was ≥ −12% for serogroups C, W-135, and Y and ≥ −15% for serogroup A (inferential analysis).

Potential statistically significant differences between groups were detected if the asymptotic standardised 95% CI for the difference in rates (percentages of participants with titres/concentrations above proposed cut-offs or with vaccine responses) between the two vaccine groups did not contain the value “0”, or if the 95% CI for the GMTs/GMCs ratios between groups did not contain the value “1” (exploratory analyses). The GMTs/GMCs ratios were computed by an analysis of covariance model on the log_10_ transformation of the titres/concentrations using the pre-vaccination log_10_ transformation of the titres/concentrations, the age strata, and the vaccine groups as covariates. No adjustment for multiplicity of secondary endpoints was made and significant results from the exploratory analyses should be interpreted with caution.

Non-inferiority of the MenACWY-TT vaccine over the MenACWY polysaccharide vaccine in terms of incidence of grade 3 general symptoms was demonstrated if the upper limit (UL) of the asymptotic 95% CI for the difference between the ACWY-TT and the Men-PS groups in the incidence of any grade 3 general symptom within four days after vaccination was below 5% (inferential analysis).

The percentages of participants reporting each solicited local and general symptom and each unsolicited AE were tabulated with exact 95% CIs. NOCIs, SAEs, and withdrawals due to AEs were described in detail.

The statistical analyses were performed using the SAS® software version 9.1 (SAS Institute Inc., Cary, NC, United States) and StatXact 7.0.

## Results

### Study participants

**Figure 1:**
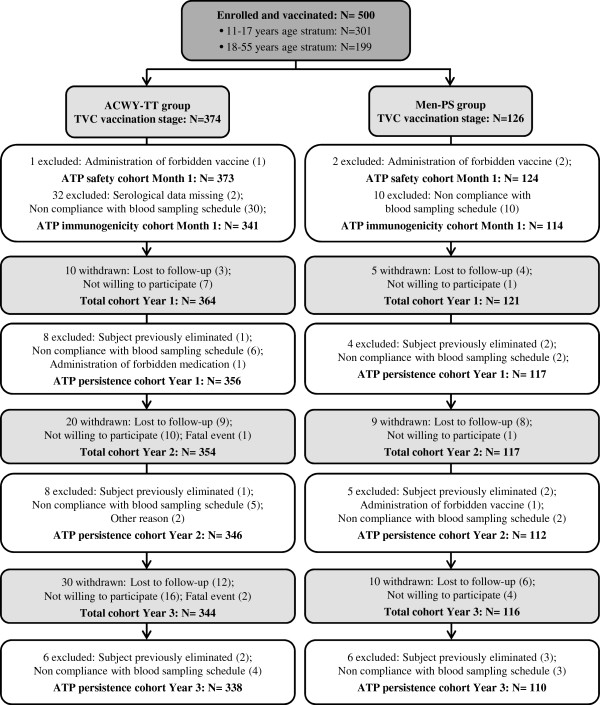
**Participant progression through the study.** ACWY-TT = group of participants who received one dose of MenACWY-TT at Month 0. Men-PS = group of participants who received one dose of the MenACWY polysaccharide vaccine at Month 0. ATP = according to protocol. TVC = total vaccinated cohort. N = number of participants. The numbers of participants withdrawn are given between the TVC vaccination stage and the total cohort for each timepoint during the persistence phase (Year 1, Year 2, and Year 3).

A total of 500 participants were enrolled and vaccinated in this study; 374 in the ACWY-TT group (225 in the 11–17 years age stratum and 149 in the 18–55 years age stratum) and 126 in the Men-PS group (76 in the 11–17 years age stratum and 50 in the 18–55 years age stratum). Of these, 485, 471, and 460 participants returned for the Year 1, Year 2, and Year 3 visits, respectively (Figure 1).

The demographic characteristics of the participants in the total vaccinated cohort were comparable for the two groups (Table 1). The mean age was 18.8 years, 45.6% of the participants were female, and the population was predominantly of South East Asian (79.8%) or of Arabic/North African (20.0%) heritage.

**Table 1:** **Summary of participant**’**s demographic characteristics at the time of vaccination**

**Characteristic**		**ACWY-TT**	**Men-PS**
		**N = 374**	**N = 126**
**Age at Month 0** (**years**)	Mean ± SD	18.6 ± 7.62	19.3 ± 8.58
	Range	11–53	11–49
**Gender**	Female n (%)	168 (44.9)	60 (47.6)
	Male n (%)	206 (55.1)	66 (52.4)
**Race**	South East Asian heritage n (%)	298 (79.7)	101 (80.2)
	Arabic/North African heritage n (%)	75 (20.1)	25 (19.8)
	East Asian heritage n (%)	1 (0.3)	0 (0.0)

ACWY-TT = group of participants who received one dose of MenACWY-TT at Month 0.

Men-PS = group of participants who received one dose of the MenACWY polysaccharide vaccine at Month 0.

N = number of participants with the administered dose (Total Vaccinated Cohort).

n (%) = number (percentage) of participants with the specified characteristic.

SD = standard deviation.

### Immunogenicity

#### Bactericidal antibodies

Since the LL of the 95% CI on the difference between the ACWY-TT and the Men-PS groups in the percentage of participants with an rSBA vaccine response was above −12% for serogroups C, W-135, and Y and above −15% for serogroup A, the primary objective of non-inferiority of the vaccine response induced by the MenACWY-TT vaccine over the MenACWY polysaccharide vaccine was met for each serogroup (Table 2). The rSBA vaccine response rates for each of the four serogroups ranged from 82.7% to 96.3% in the ACWY-TT group and from 69.7% to 91.7% in the Men-PS group. Exploratory analyses showed that the rSBA vaccine response rates for MenA and MenY were statistically significantly higher after administration of the MenACWY-TT vaccine compared with the MenACWY polysaccharide vaccine for the participants aged 11–55 years. Vaccine response rates per age strata are presented in Additional file 1: Table S1.

**Table 2:** **Differences in rSBA vaccine response rate between the ACWY**-**TT and the Men**-**PS groups**

**Antibody**	**ACWY**-**TT**		**Men**-**PS**		**Difference in vaccine response rate** (**ACWY**-**TT minus Men**-**PS**)
	**N**	**%**	**N**	**%**	**%** [**95% CI**]
rSBA-MenA	289	82.7	99	69.7	13.00 [**3**.**52**, 23.50]
rSBA-MenC	324	94.4	113	90.3	4.18 [−**1**.**03**, 11.36]
rSBA-MenW-135	326	96.3	109	91.7	4.58 [−**0**.**07**, 11.49]
rSBA-MenY	329	93.0	113	85.0	8.05 [**1**.**72**, 16.17]

ACWY-TT = group of participants who received one dose of MenACWY-TT at Month 0.

Men-PS = group of participants who received one dose of the MenACWY polysaccharide vaccine at Month 0.

Vaccine response defined as:

• rSBA titre ≥32 at Month 1 for initially seronegative participants (rSBA titre <8).

• rSBA titre at Month 1 ≥4-fold the antibody titre at Month 0 for initially seropositive participants (rSBA titre ≥8).

N = number of participants with pre- and post-vaccination results available (ATP immunogenicity cohort).

% = percentage of participants with a vaccine response.

95% CI = standardised asymptotic 95% confidence interval.

**Bold** = lower limits of the 95% confidence intervals of the differences in vaccine response rates, which were used to demonstrate non-inferiority.

At Month 1, at least 99.7% and 99.4% of the recipients of the MenACWY-TT vaccine reached rSBA titres ≥8 and ≥128 for each of the four serogroups (Table 3). In both groups, rSBA GMTs were at least 9-fold higher at Month 1 compared with pre-vaccination. The results in each of the two age strata were consistent with the results on the overall study population (Additional file 2: Table S2). Exploratory analyses did not suggest any statistically significant difference between the two groups in the percentages of participants with rSBA titres ≥8 and ≥128, but showed that rSBA GMTs adjusted for pre-vaccination titres and age strata (in the overall analysis) were higher after vaccination with MenACWY-TT compared with the MenACWY polysaccharide vaccine for all four serogroups.

**Table 3:** **Percentage of participants with rSBA titres** ≥**8 and** ≥**128 and GMTs before and after vaccination**

**Group**	**Timing**	**N**	**%** ≥**8** [**95% CI**]	**%** ≥**128** [**95% CI**]	**GMT** [**95% CI**]
**rSBA**-**MenA**					
ACWY-TT	M0	305	95.1 [92.0, 97.2]	89.5 [85.5, 92.7]	330.7 [285.8, 382.7]
	M1	323	100 [98.9, 100]	99.7 [98.3, 100]	4944.6* [4451.5, 5492.5]
	Y1	354	99.7 [98.4, 100]	99.4 [98.0, 99.9]	2084.9* [1888.3, 2302.0]
	Y2	338	99.7 [98.4, 100]	99.1* [97.4, 99.8]	1326.8* [1197.7, 1469.7]
	Y3	322	100 [98.9, 100]	99.1* [97.3, 99.8]	1238.4* [1126.0, 1361.9]
Men-PS	M0	101	87.1 [79.0, 93.0]	80.2 [71.1, 87.5]	227.8 [162.1, 320.2]
	M1	112	100 [96.8, 100]	100 [96.8, 100]	2190.1 [1857.5, 2582.2]
	Y1	113	100 [96.8, 100]	99.1 [95.2, 100]	1099.1 [931.6, 1296.7]
	Y2	102	99.0 [94.7, 100]	96.1 [90.3, 98.9]	698.9 [561.2, 870.4]
	Y3	104	100 [96.5, 100]	94.2 [87.9, 97.9]	596.9 [488.5, 729.3]
**rSBA**-**MenC**					
ACWY-TT	M0	324	78.1 [73.2, 82.5]	53.1 [47.5, 58.6]	84.1 [68.5, 103.4]
	M1	341	99.7 [98.4, 100]	99.7 [98.4, 100]	10073.7* [8699.9, 11664.5]
	Y1	353	99.7 [98.4, 100]	97.2 [94.9, 98.6]	1848.6 [1620.3, 2109.2]
	Y2	345	99.4 [97.9, 99.9]	96.2 [93.6, 98.0]	1162.0 [1013.1, 1332.9]
	Y3	337	99.1 [97.4, 99.8]	92.9 [89.6, 95.4]	870.3 [757.1, 1000.4]
Men-PS	M0	113	85.0 [77.0, 91.0]	49.6 [40.0, 59.1]	114.1 [80.3, 162.0]
	M1	114	100 [96.8, 100]	98.2 [93.8, 99.8]	6545.6 [5047.5, 8488.4]
	Y1	115	99.1 [95.3, 100]	95.7 [90.1, 98.6]	1876.5 [1400.7, 2514.0]
	Y2	112	98.2 [93.7, 99.8]	92.0 [85.3, 96.3]	1229.4 [876.4, 1724.7]
	Y3	109	99.1 [95.0, 100]	93.6 [87.2, 97.4]	1124.8 [812.3, 1557,6]
**rSBA**-**MenW**-**135**					
ACWY-TT	M0	327	75.5 [70.5, 80.1]	58.4 [52.9, 63.8]	93.0 [74.6, 116.0]
	M1	340	99.7 [98.4, 100]	99.4 [97.9, 99.9]	8576.5* [7614.9, 9659.5]
	Y1	356	99.7 [98.4, 100]	99.4* [98.0, 99.9]	2993.5* [2618.8, 3421.9]
	Y2	346	99.4 [97.9, 99.9]	98.6* [96.7, 99.5]	1984.6* [1757.1, 2241.4]
	Y3	336	99.7* [98.4, 100]	98.8* [97.0, 99.7]	2109.2* [1842.5, 2414.5]
Men-PS	M0	109	81.7 [73.1, 88.4]	61.5 [51.7, 70.6]	115.3 [81.6, 162.9]
	M1	114	100 [96.8, 100]	100 [96.8, 100]	2969.5 [2439.4, 3614.9]
	Y1	117	100 [96.9, 100]	94.9 [89.2, 98.1]	699.9 [569.3, 860.4]
	Y2	111	90.1 [83.0, 94.9]	81.1 [72.5, 87.9]	319.1 [228.0, 446.5]
	Y3	105	86.7 [78.6, 92.5]	80.0 [71.1, 87.2]	332.8 [224.4, 493.8]
**rSBA**-**MenY**					
ACWY-TT	M0	330	92.7 [89.4, 95.3]	80.0 [75.3, 84.2]	310.0 [261.2, 367.9]
	M1	340	100 [98.9, 100]	99.7 [98.4, 100]	10315.2* [9317.1, 11420.2]
	Y1	355	100 [99.0, 100]	99.7 [98.4, 100]	4207.1* [3767.3, 4698.3]
	Y2	345	99.7 [98.4, 100]	99.1* [97.5, 99.8]	3042.1* [2692.2, 3437.5]
	Y3	338	99.7 [98.4, 100]	99.4 [97.9, 99.9]	2567.3* [2288.6, 2879.8]
Men-PS	M0	113	92.9 [86.5, 96.9]	77.9 [69.1, 85.1]	282.8 [209.9, 380.8]
	M1	114	100 [96.8, 100]	100 [96.8, 100]	4573.7 [3863.9, 5413.9]
	Y1	116	100 [96.9, 100]	98.3 [93.9, 99.8]	1386.5 [1104.2, 1740.9]
	Y2	111	99.1 [95.1, 100]	94.6 [88.6, 98.0]	850.2 [667.5, 1082.9]
	Y3	108	99.1 [94.9, 100]	97.2 [92.1, 99.4]	848.0 [682.6, 1053.5]

ACWY-TT = group of participants who received one dose of MenACWY-TT at Month 0.

Men-PS = group of participants who received one dose of the MenACWY polysaccharide vaccine at Month 0.

N = number of participants with available results.

% = percentage of participants with titres within the specified range.

GMT = geometric mean titre.

95% CI = 95% confidence interval.

M0 = pre-vaccination blood sample taken at Month 0; M1 = post-vaccination blood sample taken at Month 1 (ATP immunogenicity cohort); Y1 = post-vaccination blood sample taken at Year 1 (ATP cohort for persistence Year 1); Y2 = post-vaccination blood sample taken at Year 2 (ATP cohort for persistence Year 2); Y3 = post-vaccination blood sample taken at Year 3 (ATP cohort for persistence Year 3).

^*^Statistically significantly higher value in the ACWY-TT group compared to the Men-PS group in exploratory analyses.

#### Persistence of the bactericidal antibodies

**Figure 2:**
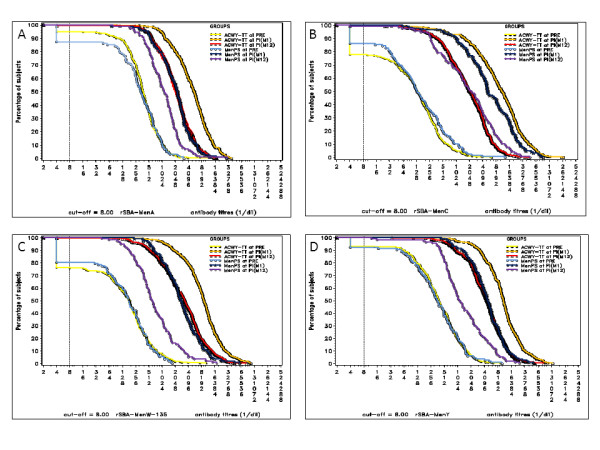
**Reverse cumulative curves for the ACWY**-**TT and the Men**-**PS groups for rSBA**-**MenA** (**A**), **rSBA**-**MenC** (**B**), **rSBA**-**MenW**-**135** (**C**), **and rSBA**-**MenY** (**D.**) ACWY-TT = group of participants who received one dose of MenACWY-TT at Month 0. Men-PS = group of participants who received one dose of the MenACWY polysaccharide vaccine at Month 0. PRE = pre-vaccination at Month 0; PI(M1) = post-vaccination at Month 1 (ATP immunogenicity cohort); PI(M12) = post-vaccination at Month 12 (ATP cohort for persistence Year 1).

At Year 3, ≥99.1% and ≥92.9% of the participants in the ACWY-TT group and ≥86.7% and ≥80.0% in the MenPS group retained rSBA titres ≥8 and ≥128 for each of the four serogroups (Table 3). In both groups, the rSBA GMTs at Year 3 remained higher than the values observed at Month 0. The rSBA GMTs decay rates were highest for all four serogroups between Month 1 and Year 1 (from 2.4- to 5.4-fold in the ACWY-TT group and from 2.0- to 4.2-fold in the Men-PS group) (Figure 2). Lower rSBA GMTs decay rates for all four serogroups were observed between Year 1 and Year 2 (from 1.4- to 1.6-fold in the ACWY-TT group and from 1.5- to 2.2-fold in the Men-PS group) and between Year 2 and Year 3 (from 0.9- to 1.3-fold in the ACWY-TT group and from 1.0- to 1.2-fold in the Men-PS group).

Exploratory analyses showed that rSBA GMTs adjusted for pre-vaccination titres and age strata for serogroups A, W-135, and Y were statistically significantly higher after vaccination with MenACWY-TT compared with the MenACWY polysaccharide vaccine up to Year 3 (Additional file 2: Table S2).

#### Anti-tetanus antibodies

At Month 1, the percentage of participants with anti-TT concentrations ≥0.1 IU/mL increased from 67.4% (95% CI: 62.0–72.4) to 95.6% (95% CI: 92.8–97.5) in the ACWY-TT group and a 28-fold increase of the anti-TT GMCs was observed in this group (from 0.35 [95% CI: 0.29 – 0.43] at Month 0 to 10.01 [95% CI: 8.34 – 12.03] at Month 1). As expected, the percentage of participants with anti-TT concentrations ≥0.1 IU/mL (60.0% [95% CI: 50.2-69.2] at Month 0 and 56.6% [95% CI: 47.0–65.9] at Month 1) and the anti-TT GMCs (0.28 [95% CI: 0.20–0.39] at Month 0 and 0.27 [95% CI: 0.19–0.38] at Month 1) remained stable between pre- and post-vaccination in the Men-PS group.

### Safety/reactogenicity

During the four-day post-vaccination period, grade 3 general symptoms were reported by five participants (1.3%) in the ACWY-TT group and by no participant in the Men-PS group (Table 4). The difference between the ACWY-TT and the Men-PS groups in the incidence of grade 3 general symptoms was 1.34% (95% CI: -1.64 – 3.09). The non-inferiority of the MenACWY-TT vaccine over the MenACWY polysaccharide vaccine in terms of incidence of any grade 3 general symptom was demonstrated, as the UL of the standardised asymptotic 95% CI was below the pre-specified non-inferiority limit of 5%. Any grade 3 symptoms were reported by 12 participants (3.2%) in the ACWY-TT group and by one participant (0.8%) in the Men-PS group (Table 4).

**Table 4:** Difference between groups in the percentage of participants reporting grade 3 solicited and unsolicited symptoms

**Symptoms**	**ACWY**-**TT**			**Men**-**PS**			**Difference in percentage** (**ACWY**-**TT minus Men**-**PS**)
	**N**	**n**	**%**	**N**	**n**	**%**	**%** [**95% CI**]
Any symptom	374	12	3.2	126	1	0.8	2.41 [−1.29, 4.91]
General symptom	374	5	1.3	126	0	0.0	1.34 [−1.64, **3**.**09**]
Local symptom	374	9	2.4	126	1	0.8	1.61 [−2.06, 3.90]

ACWY-TT = group of participants who received one dose of MenACWY-TT at Month 0.

Men-PS = group of participants who received one dose of the MenACWY polysaccharide vaccine at Month 0.

N = number of participants with the administered dose (total vaccinated cohort).

n/% = number/percentage of participants reporting a specified symptom during the four-day post-vaccination period.

95% CI = standardised asymptotic 95% confidence interval.

**Bold** = upper limits of the 95% confidence intervals of the differences in percentages of participants reporting grade 3 general symptoms, which were used to demonstrate non-inferiority (co-primary objective).

**Figure 3:**
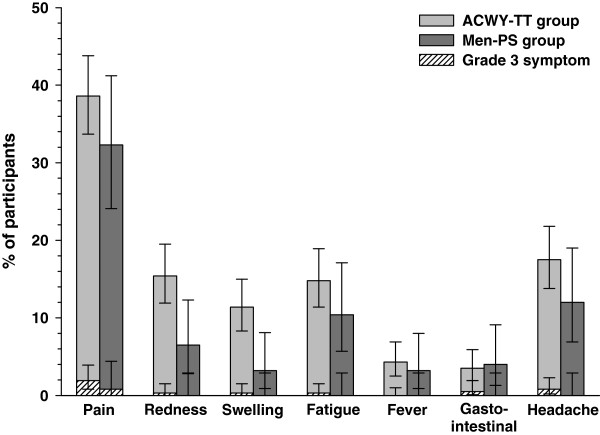
**Percentage of participants in the ACWY**-**TT and Men**-**PS groups experiencing solicited local and general symptoms( total vaccinated cohort).** ACWY-TT = group of participants who received one dose of MenACWY-TT at Month 0. Men-PS = group of participants who received one dose of the MenACWY polysaccharide vaccine at Month 0.% = percentage of participants with at least once the symptom within the four-day post-vaccination period (total vaccinated cohort). Error bars represent 95% confidence intervals.

During the four-day follow-up after vaccination, pain was the most frequently reported solicited local symptom in both groups (in 143/370 [38.6%] participants in the ACWY-TT group and 40/124 [32.3%] participants in the Men-PS group). The most common solicited general symptom was headache in both groups, which was reported by 65/371 (17.5%) participants in the ACWY-TT group and 15/125 (12.0%) participants in the Men-PS group. Injection site redness and swelling were more frequently reported in the ACWY-TT group (15. 4% and 11.4% respectively) than in the Men-PS group (6.5% and 3.2% respectively) (Figure 3). Grade 3 solicited symptoms were infrequently reported in both groups and no participants reported grade 3 fever (oral temperature ≥39.5°C).

During the 6-month extended safety follow-up period, one participant reported a NOCI (food allergy) and one participant reported two SAEs (costochondritis and mental disorder). The SAEs were reported in the ACWY-TT group, but none were considered related to vaccination by the investigators. No participants reported NOCIs or SAEs that were considered related to vaccination or events related to the lack of vaccine efficacy up to three years after vaccination. In the persistence phase of the study, two participants of the ACWY-TT group died due to a traffic accident (one subject between Year 1 and Year 2 and a second subject between Year 2 and Year 3).

## Discussion

Non-inferiority of the immunogenicity of the MenACWY-TT vaccine as compared to the MenACWY polysaccharide vaccine in terms of rSBA vaccine response to the four meningococcal serogroups was demonstrated. At one month post-vaccination, rSBA titres ≥128, which is the conservative threshold for protection against meningococcal diseases, were reached for each of the four serogroups by at least 99.4% and 98.2% of the participants vaccinated with MenACWY-TT and the MenACWY polysaccharide vaccine, respectively. This observation is consistent with the results of previous studies in which the immune response induced by MenACWY-TT was evaluated in various age groups [28-34]. Moreover, the percentage of participants who retained rSBA antibody titres ≥8 up to three years after vaccination remained high for the four serogroups in both groups, indicating that the protection continues for at least three years in adolescents and adults. The high percentage (92.9%) of participants who retained rSBA titres ≥128 in the present study contrasts with previous observations, showing waning immunity in terms of hSBA titres and concerns about the potential for vaccine failure following vaccination with either the MenACWY polysaccharide vaccine or another quadrivalent meningococcal conjugate vaccine using diphtheria toxoid as carrier protein (MenACWY-DT) [40]. However, the two studies cannot be directly compared since a different assay using human complement (hSBA) was used in the study with MenACWY-DT.

Exploratory analyses showed that the rSBA GMTs for each meningococcal serogroup were statistically significantly higher at one month post-vaccination in participants vaccinated with MenACWY-TT compared with those who received the MenACWY polysaccharide vaccine, although no differences were detected in terms of percentage of participants with rSBA titres ≥8 or ≥128. These results are consistent with those of previous studies conducted on the MenACWY-TT vaccine in children 3–5 years of age [29] and in adolescents and young adults [28] in Europe, and in children [33] and adolescents [34] in Asia. These results are also in-line with other studies showing that other quadrivalent meningococcal conjugate vaccines (MenACWY-DT and MenACWY-CRM_197_) induce higher antibody titres than plain polysaccharide vaccines in children, adolescents, and adults [25,26,41-43]. The rSBA GMTs for serogroups A, W-135, and Y remained statistically significantly higher in the participants who received the MenACWY-TT vaccine than in those who received the MenACWY polysaccharide vaccine at three years post-vaccination, while comparable rSBA GMTs for serogroup C were measured in both vaccine groups. The antibody decay for each serogroup was highest between one month and one year post-vaccination and the rate of decay decreased at subsequent timepoints in both groups. These data indicate that decay over time may lead to the need for further booster doses if the individual remains at risk for meningococcal disease. The evaluation of additional timepoints in the current study will further contribute to the understanding of the expected long-term duration of protection.

In this study, high rSBA titres were measured in both groups at pre-vaccination. This was expected for the age groups studied (adolescents and adults) because immunity to meningococcal strains is known to increase in an age-dependent manner [44]. Moreover, the likelihood of exposure to *N*. *meningitidis* and acquisition of functional antibodies due to natural immunity may vary by location as meningococcal disease epidemiology varies substantially by geographic area [3]. Vaccine response rate was chosen as the primary endpoint to evaluate the immunogenicity of the vaccines as vaccine response assesses the ability of participants to respond to the vaccine regardless of their serostatus at pre-vaccination. As expected, immune responses to tetanus toxoid were observed in the participants who received the MenACWY-TT vaccine, but not in those who received the MenACWY polysaccharide vaccine [28,29,33,34].

Non-inferiority of the MenACWY-TT vaccine over the MenACWY polysaccharide vaccine, in terms of incidence of solicited and unsolicited grade 3 general symptoms reported within four days after vaccination, was demonstrated. Of note, clinically relevant severe general symptoms and vaccine-related events are most likely to occur during the four-day post-vaccination period. However, the incidence of any grade 3 symptoms seemed higher in the participants who received the MenACWY-TT vaccine than the MenACWY polysaccharide vaccine and this observation was driven by the local injection site reactions. The specific solicited injection site redness and swelling with any intensity were also more frequently reported in the participants who received the MenACWY-TT vaccine than the MenACWY polysaccharide vaccine. These observations are likely due to the TT content of the conjugate vaccine and are consistent with previous studies showing that local reactions are more frequent in individuals vaccinated with quadrivalent meningococcal conjugate vaccines compared to plain polysaccharide vaccines [27-29,33,34,43]. However, we cannot exclude that the intramuscular administration of the conjugate vaccine (versus subcutaneous for the polysaccharide one) may in part explain the higher reactogenicity as a previous study suggested that intramuscular administration of meningococcal polysaccharide vaccine induced higher rates of erythema compared to subcutaneous administration [45]. No NOCIs or SAEs reported up to three years after vaccination were considered related to vaccination in this study and both vaccines were well-tolerated.

The present study was limited by its open design due to the differences in the appearance and administration mode of the two study vaccines. The open design would not have influenced the immunogenicity results because the laboratory personnel was blinded during serological testing but had the potential to bias the safety reporting in favour of the control plain polysaccharide vaccine versus the meningococcal conjugate vaccine. The interpretation of the results has also been limited by the absence of validated correlates of protection for serogroups A, W-135, and Y and by the numerous exploratory statistical comparisons, which should be interpreted with caution. The generalizability of our results was limited by the small sample size and by the fact that the study was conducted in two countries. Other studies are being conducted in parallel to evaluate the immunogenicity and safety of the MenACWY-TT vaccine in younger age groups and to compare this vaccine with other licensed quadrivalent meningococcal conjugate vaccines [30]. Of note, at the time of study conduct, meningococcal conjugate vaccines were not licensed in Saudi Arabia or the Philippines.

## Conclusion

This study showed that the MenACWY-TT vaccine, when administered as a single intramuscular dose to adolescents and adults 11–55 years of age, was non-inferior to a licensed meningococcal polysaccharide vaccine in terms of both immunogenicity and safety endpoints. Furthermore, MenACWY-TT induced an immune response against meningococcal serogroups A, C, W-135, and Y, which persisted up to three years after vaccination; rSBA GMTs for serogroups A, W-135, and Y were statistically significantly higher compared with those in participants who received the MenACWY polysaccharide vaccine, while comparable rSBA GMTs for serogroup C were measured in both vaccine groups. Both vaccines were well-tolerated and their safety and reactogenicity profiles were comparable, except for higher reported rates of injection site redness and swelling after vaccination with MenACWY-TT.

*Mencevax* and *Nimenrix* are trademark of the GlaxoSmithKline Group of companies.

## Abbreviations

ATP: According to protocol; CI: Confidence interval; ELISA: Enzyme-linked immunosorbent assay; GMT: Geometric mean antibody titres; GMC: Geometric mean antibody concentration; LL: Lower limit; UL: Upper limit; MenACWY-TT: Meningococcal quadrivalent serogroups A, C,W-135 and Y vaccine with all serogroups conjugated to the tetanus toxoid (TT) carrier protein; Men-PS: Meningococcal quadrivalent polysaccharide vaccine; NOCI: New onset of chronic illness; rSBA: Serum bactericidal antibody assays using baby rabbit serum as exogenous complement source; (S)AE: (Serious) adverse event; TT: Tetanus toxoid.

## Competing interests

The institutes of Dr Charissa Borja-Tabora (*Research Institute for Tropical Medicine*, *Philippines*) and Dr Ziad A. Memish (*King Abdulaziz Medical City National Guard Health affairs in 2006*) received grants from the GlaxoSmithKline group of companies. Dr Charissa Borja-Tabora received support for meetings, travel or accommodation expenses from the GlaxoSmithKline group of companies in the past 5 years. Veronique Bianco and Drs Marie Van der Wielen, Dominique Boutriau and Jacqueline Miller are employees of GlaxoSmithKline group of companies. Drs Marie Van der Wielen and Dominique Boutriau declare stock ownership in the GlaxoSmithKline group of companies. Dr Jacqueline Miller declares restricted share in the GlaxoSmithKline group of companies. Dr Dominique Boutriau is also inventor of certain patents of the GlaxoSmithKline group of companies. Dr Cecilia Montalban declares that she has no competing interests.

## Authors’ contributions

Drs CB-T, CM and ZAM were principal investigators and were involved in the recruitment of investigators, the supervision of the study, administrative, logistic and technical supports, the collection of the data, and the drafting and approval of the manuscript. Drs MVdW, DB, JM (clinical development managers) and VB (biostatistician) were employed by GlaxoSmithKline group of companies and involved in all stages of the study (study design, data analyses and interpretations, drafting and approval of the manuscript). All authors read and approved the final manuscript.

## Pre-publication history

The pre-publication history for this paper can be accessed here:

http://www.biomedcentral.com/1471-2334/13/116/prepub

## Supplementary Material

Additional file 1: Table S1 Percentage of participants per age strata with an rSBA vaccine response. Click here for file

Additional file 2: Table S2 Percentage of participants per age strata with rSBA titres ≥1:8 and 1:128 and GMTs. Click here for file
