# Supplementary material for: Immune response, antibody persistence, and safety of a single dose of the quadrivalent meningococcal serogroups A, C, W-135, and Y tetanus toxoid conjugate vaccine in adolescents and adults: results of an open, randomised, controlled study
Source: BMC Infect Dis. 2013 Mar 5;13:116. doi: 10.1186/1471-2334-13-116 (PMC3599520; doi:10.1186/1471-2334-13-116)
Supplement: Additional file 1: Table S1 — Percentage of participants per age strata with an rSBA vaccine response. [file 1471-2334-13-116-S1.pdf]

Supplementary Table 1 Percentage of participants per age strata with an rSBA vaccine response.

	11–17 years age stratum				18–55 years age stratum			
**Antibody**	**ACWY-TT**		**Men-PS**		**ACWY-TT**		**Men-PS**	
	**N**	**% (95% CI)**	**N**	**%**	**N**	**%**	**N**	**%**
rSBA-MenA	197	81.7 [75.6, 86.9]	65	70.8 [58.2, 81.4]	92	84.8 [75.8, 91.4]	34	67.6 [49.5, 82.6]
rSBA-MenC	210	96.7 [93.3, 98.6]	75	88.0 [78.4, 94.4]	114	90.4 [83.4, 95.1]	38	94.7 [82.3, 99.4]
rSBA-MenW-135	215	96.7 [93.4, 98.7]	72	91.7 [82.7, 96.9]	111	95.5 [89.8, 98.5]	37	91.9 [78.1, 98.3]
rSBA-MenY	216	94.0 [89.9, 96.8]	75	82.7 [72.2, 90.4]	113	91.2 [84.3, 95.7]	38	89.5 [75.2, 97.1]

Footnote: ACWY-TT= group of participants who received one dose of MenACWY-TT at Month 0

Men-PS= group of participants who received one dose of the MenACWY polysaccharide vaccine at Month 0

Vaccine response defined as:

- For initially seronegative participants: antibody titre ≥1:32 at Month 1
- For initially seropositive participants: antibody titre at Month 1 ≥4-fold the antibody titre at Month 0

N= number of participants with pre- and post-vaccination results available (ATP immunogenicity cohort)

%= percentage of participants with a vaccine response

95% CI= standardised asymptotic 95% confidence interval
