# Supplementary material for: Immune response, antibody persistence, and safety of a single dose of the quadrivalent meningococcal serogroups A, C, W-135, and Y tetanus toxoid conjugate vaccine in adolescents and adults: results of an open, randomised, controlled study
Source: BMC Infect Dis. 2013 Mar 5;13:116. doi: 10.1186/1471-2334-13-116 (PMC3599520; doi:10.1186/1471-2334-13-116)
Supplement: Additional file 2: Table S2 — Percentage of participants per age strata with rSBA titres ≥1:8 and 1:128 and GMTs. [file 1471-2334-13-116-S2.pdf]

Supplementary Table 2 Percentage of participants per age strata with rSBA titres ≥1:8 and 1:128 and GMTs

Footnote: ACWY-TT= group of participants who received one dose of MenACWY-TT at Month 0

Men-PS= group of participants who received one dose of the MenACWY polysaccharide vaccine at Month 0

N = number of participants with available results

%= percentage of participants with titres within the specified range

GMT = geometric mean titre

95% CI = 95% confidence interval

M0=pre-vaccination blood sample taken at Month 0; M1= post-vaccination blood sample taken at Month 1 (ATP immunogenicity cohort); Y1=post-vaccination blood sample taken at Year 1 (ATP cohort for persistence Year 1); Y2=post-vaccination blood sample taken at Year 2 (ATP cohort for persistence Year 2); Y3=post-vaccination blood sample taken at Year 3 (ATP cohort for persistence Year 3).

		11–17 years age stratum				18–55 years age stratum			
**Group**	**Timing**	**N**	**% ≥1:8 [95% CI]**	**% ≥1:128 [95% CI]**	**GMT [95% CI]**	**N**	**% ≥1:8 [95% CI]**	**% ≥1:128 [95% CI]**	**GMT [95% CI]**
**rSBA-MenA**									
ACWY-TT	M0	205	96.6 [93.1, 98.6]	92.7 [88.2, 95.8]	379.1 [323.6, 444.0]	100	92.0 [84.8, 96.5]	83.0 [74.2, 89.8]	250.0 [184.8, 338.1]
	M1	214	100 [98.3, 100]	100 [98.3, 100]	5484.1 [4832.4, 6223.6]	109	100 [96.7, 100]	99.1 [95.0, 100]	4035.0 [3354.9, 4853.1]
	Y1	218	100 [98.3, 100]	99.5 [97.5, 100]	2369.1 [2111.5, 2658.0]	136	99.3 [96.0, 100]	99.3 [96.0, 100]	1698.8 [1423.9, 2026.9]
	Y2	216	100 [98.3, 100]	100 [98.3, 100]	1501.1 [1335.1, 1687.7]	122	99.2 [95.5, 100]	97.5 [93.0, 99.5]	1066.4 [882.5, 1288.6]
	Y3	215	100 [98.3, 100]	99.5 [97.4, 100]	1412.4 [1265.2, 1576.7]	107	100 [96.6, 100]	98.1 [93.4, 99.8]	950.8 [799.5, 1130.9]
Men-PS	M0	67	92.5 [83.4, 97.5]	88.1 [77.8, 94.7]	319.9 [223.9, 456.9]	34	76.5 [58.8, 89.3]	64.7 [46.5, 80.3]	116.7 [57.9, 235.2]
	M1	74	100 [95.1, 100]	100 [95.1, 100]	2640.8 [2180.6, 3198.0]	38	100 [90.7, 100]	100 [90.7, 100]	1521.3 [1140.7, 2028.8]
	Y1	75	100 [95.2, 100]	100 [95.2, 100]	1211.4 [986.8, 1487.3]	38	100 [90.7, 100]	97.4 [86.2, 99.9]	907.1 [684.5, 1202.0]
	Y2	67	98.5 [92.0, 100]	98.5 [92.0, 100]	808.0 [618.2 1056.2]	35	100 [90.0, 100]	91.4 [76.9, 98.2]	529.4 [360.8, 776.9]
	Y3	70	100 [94.9, 100]	97.1 [90.1, 99.7]	721.5 [565.7, 920.2]	34	100 [89.7, 100]	88.2 [72.5, 96.7]	404.1 [290.0, 563.0]
**rSBA-MenC**									
ACWY-TT	M0	210	75.2 [68.8, 80.9]	50.5 [43.5, 57.4]	75.2 [57.9, 97.6]	114	83.3 [75.2, 89.7]	57.9 [48.3, 67.1]	103.5 [74.0, 144.9]
	M1	224	99.6 [97.5, 100]	99.6 [97.5, 100]	10727.4 [9017.9, 12761.0]	117	100 [96.9, 100]	100 [96.9, 100]	8931.3 [6811.3, 11711.0]
	Y1	218	99.5 [97.5, 100]	97.7 [94.7, 99.3]	1966.5 [1660.4, 2329.0]	135	100 [97.3, 100]	96.3 [91.6, 98.8]	1673.1 [1353.6, 2068.0]
	Y2	220	99.5 [97.5, 100]	97.7 [94.8, 99.3]	1141.2 [974.2, 1336.9]	125	99.2 [95.6, 100]	93.6 [87.8, 97.2]	1199.6 [925.5, 1554.9]
	Y3	221	99.1 [96.8, 99.9]	92.8 [88.5, 95.8]	902.3 [759.8, 1071.4]	116	99.1 [95.3, 100]	93.1 [86.9, 97.0]	812.6 [638.5, 1034.0]
Men-PS	M0	75	78.7 [67.7, 87.3]	50.7 [38.9, 62.4]	105.4 [65.5, 169.5]	38	97.4 [86.2, 99.9]	47.4 [31.0, 64.2]	133.4 [82.3, 216.2]
	M1	76	100 [95.3, 100]	97.4 [90.8, 99.7]	6112.2 [4352.7, 8582.9]	38	100 [90.7, 100]	100 [90.7, 100]	7506.8 [5026.3, 11211.5]
	Y1	73	98.6 [92.6, 100]	97.3 [90.5, 99.7]	1739.9 [1182.5, 2560.2]	42	100 [91.6, 100]	92.9 [80.5, 98.5]	2139.9 [1358.1, 3371.7]
	Y2	75	97.3 [90.7, 99.7]	90.7 [81.7, 96.2]	986.2 [635.8, 1529.8]	37	100 [90.5, 100]	94.6 [81.8, 99.3]	1921.9 [1160.6, 3182.4]
	Y3	76	98.7 [92.9, 100]	93.4 [85.3, 97.8]	995.0 [657.7, 1505.2]	33	100 [89.4, 100]	93.9 [79.8, 99.3]	1492.1 [891.3, 2497.7]
**rSBA-MenW-135**									
ACWY-TT	M0	216	78.2 [72.1, 83.6]	65.7 [59.0, 72.0]	118.9 [91.4, 154.7]	111	70.3 [60.9, 78.6]	44.1 [34.7, 53.9]	57.8 [39.0, 85.5]

		11–17 years age stratum				18–55 years age stratum			
**Group**	**Timing**	**N**	**% ≥1:8 [95% CI]**	**% ≥1:128 [95% CI]**	**GMT [95% CI]**	**N**	**% ≥1:8 [95% CI]**	**% ≥1:128 [95% CI]**	**GMT [95% CI]**
	M1	223	99.6 [97.5, 100]	99.1 [96.8, 99.9]	10562.9 [9179.5, 12154.9]	117	100 [96.9, 100]	100 [96.9, 100]	5765.9 [4708.8, 7060.3]
	Y1	219	99.5 [97.5, 100]	99.5 [97.5, 100]	3676.7 [3111.5, 4344.6]	137	100 [97.3, 100]	99.3 [96.0, 100]	2155.1 [1740.1, 2669.2]
	Y2	221	99.5 [97.5, 100]	99.1 [96.8, 99.9]	2209.6 [1909.5, 2556.8]	125	99.2 [95.6, 100]	97.6 [93.1, 99.5]	1641.3 [1323.4, 2035.7]
	Y3	221	99.5 [97.5, 100]	99.1 [96.8, 99.9]	2507.7 [2141.1, 2937.2]	115	100 [96.8, 100]	98.3 [93.9, 99.8]	1512.5 [1184.5, 1931.2]
Men-PS	M0	72	84.7 [74.3, 92.1]	68.1 [56.0, 78.6]	142.5 [94.4, 215.0]	37	75.7 [58.8, 88.2]	48.6 [31.9, 65.6]	76.4 [40.5, 144.1]
	M1	76	100 [95.3, 100]	100 [95.3, 100]	3548.7 [2837.0, 4438.9]	38	100 [90.7, 100]	100 [90.7, 100]	2079.4 [1431.6, 3020.2]
	Y1	75	100 [95.2, 100]	94.7 [86.9, 98.5]	773.8 [595.8, 1005.0]	42	100 [91.6, 100]	95.2 [83.8, 99.4]	584.9 [414.7, 825.0]
	Y2	74	87.8 [78.2, 94.3]	81.1 [70.3, 89.3]	300.9 [195.7, 462.7]	37	94.6 [81.8, 99.3]	81.1 [64.8, 92.0]	358.7 [206.6, 622.8]
	Y3	73	86.3 [76.2, 93.2]	79.5 [68.4,88.0]	348.9 [215.7, 564.4]	32	87.5 [71.0, 96.5]	81.3 [63.6, 92.8]	298.9 [144.7, 617.4]
**rSBA-MenY**									
ACWY-TT	M0	217	93.1 [88.9, 96.1]	85.3 [79.8, 89.7]	366.6 [297.6, 451.5]	113	92.0 [85.4, 96.3]	69.9 [60.6, 78.2]	224.6 [167.2, 301.8]
	M1	223	100 [98.4, 100]	99.6 [97.5, 100]	12536.3 [11171.5, 14068.0]	117	100 [96.9, 100]	100 [96.9, 100]	7113.0 [5929.8, 8532.2]
	Y1	218	100 [98.3, 100]	99.5 [97.5, 100]	4943.7 [4298.3, 5686.1]	137	100 [97.3, 100]	100 [97.3, 100]	3254.5 [2736.7, 3870.3]
	Y2	221	99.5 [97.5, 100]	98.6 [96.1, 99.7]	3145.3 [2689.1, 3678.9]	124	100 [97.1, 100]	100 [97.1, 100]	2866.5 [2355.6, 3488.2]
	Y3	221	99.5 [97.5, 100]	99.5 [97.5, 100]	2941.7 [2551.9, 3391.0]	117	100 [96.9, 100]	99.1 [95.3, 100]	1985.1 [1642.9, 2398.6]
Men-PS	M0	75	96.0 [88.8, 99.2]	88.0 [78.4, 94.4]	431.1 [313.4, 593.1]	38	86.8 [71.9, 95.6]	57.9 [40.8, 73.7]	123.0 [70.9, 213.4]
	M1	76	100 [95.3, 100]	100 [95.3, 100]	5103.0 [4197.2, 6204.4]	38	100 [90.7, 100]	100 [90.7, 100]	3674.1 [2659.7, 5075.4]
	Y1	74	100 [95.1, 100]	100 [95.1, 100]	1641.1 [1272.0, 2117.4]	42	100 [91.6, 100]	95.2 [83.8, 99.4]	1030.1 [662.8, 1600.9]
	Y2	74	100 [95.1, 100]	95.9 [88.6, 99.2]	949.5 [726.9, 1240.2]	37	97.3 [85.8, 99.9]	91.9 [78.1, 98.3]	681.7 [411.8, 1128.3]
	Y3	76	100 [95.3, 100]	100 [95.3, 100]	978.1 [780.3, 1226.0]	32	96.9 [83.8, 99.9]	90.6 [75.0, 98.0]	604.3 [366.1, 997.5]
